# Supplementary material for: Hydroxylated Fatty Acids: The Role of the Sphingomyelin Synthase and the Origin of Selectivity
Source: Membranes (Basel). 2021 Oct 16;11(10):787. doi: 10.3390/membranes11100787 (PMC8539438; doi:10.3390/membranes11100787)
Supplement: Supplementary file 1 [file membranes-11-00787-s001.zip › membranes-1412067-supplementary.pdf]

Supplementary materials

Hydroxylated Fatty Acids: The Role of the Sphingomyelin Synthase and the Origin of Selectivity

Lucia Sessa *, Anna Maria Nardiello, Jacopo Santoro, Simona Concilio and Stefano Piotto *

Department of Pharmacy, University of Salerno, via Giovanni Paolo II, 84084 132 Fisciano (SA), Italy; annardiello@unisa.it (A.M.N.); jsantoro@unisa.it (J.S.); sconcilio@unisa.it (S.C.)

* Correspondence: lucsessa@unisa.it (L.S.); piotto@unisa.it (S.P.)

Reconstruction of the SMS pathway

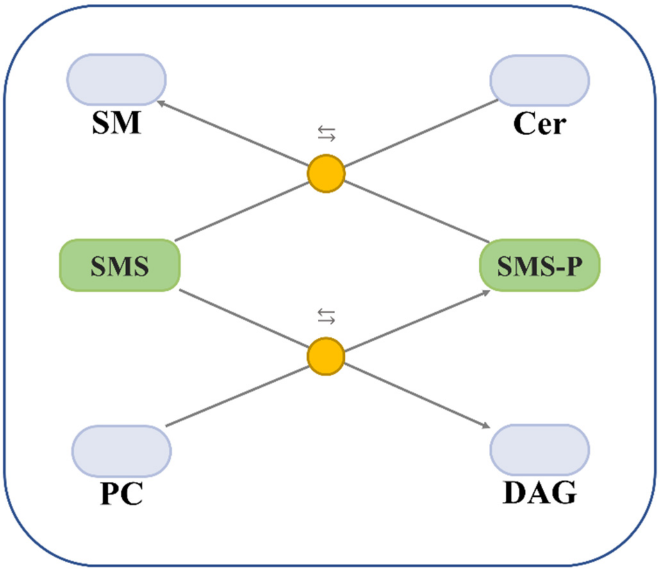

Figure S1. Graphical representation of modeled reactions. In green are the enzyme SMS and the enzyme state with the modified tyrosine (SMS-P). In gray are the substrates PC, DAG, Cr and SM.

3D-structure validation

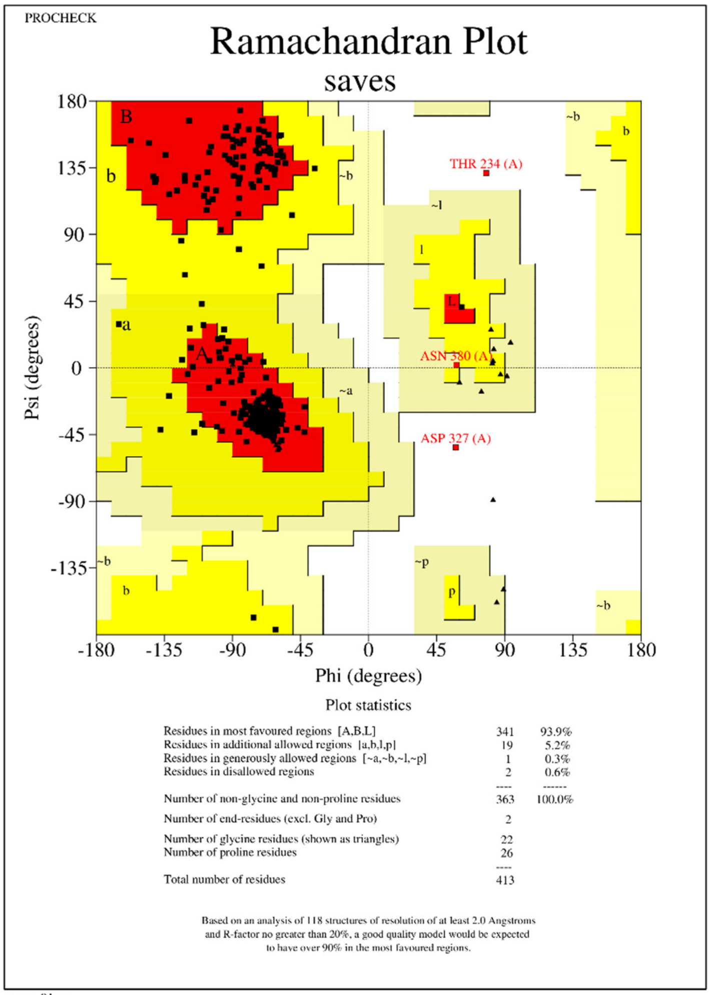

saves_01.ps

Figure S2. Ramachandran plot for SMS1 showing the presence of amino acid residues in favored, allowed, and outlier regions

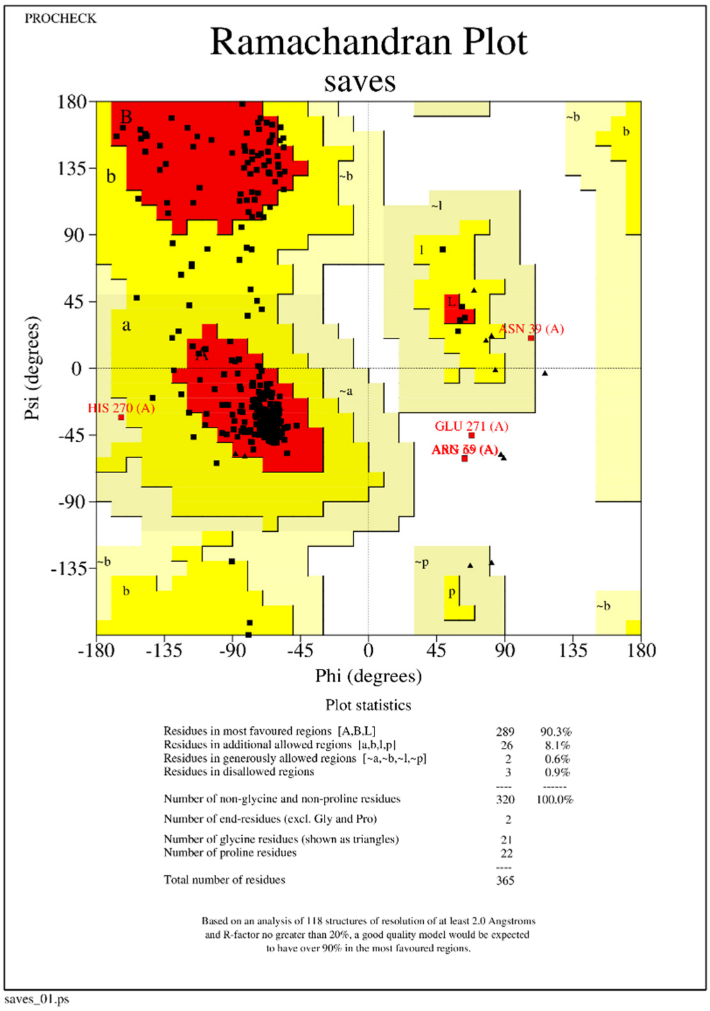

Figure S3. Ramachandran plot for SMS2 showing the presence of amino acid residues in favored, allowed, and outlier regions

Table S1. Summary of the quality of SMS1 structure

SMS1 structure built in this work	Previous SMS1 structure
Structure Z-scores, positive is better than average:	Structure Z-scores, positive is better than average:
1st generation packing quality : -1.538	1st generation packing quality : -3.250
2nd generation packing quality : -1.675	2nd generation packing quality : -3.718 (poor)
Ramachandran plot appearance : -0.541	Ramachandran plot appearance : -3.698 (poor)
chi-1/chi-2 rotamer normality : 1.311	chi-1/chi-2 rotamer normality : -3.618 (poor)
Backbone conformation : -1.966	Backbone conformation : -3.838 (poor)
RMS Z-scores, should be close to 1.0:	RMS Z-scores, should be close to 1.0:
Bond lengths : 1.127	Bond lengths : 1.174
Bond angles : 0.442 (tight)	Bond angles : 0.675
Omega angle restraints : 1.469 (loose)	Omega angle restraints : 1.655 (loose)
Side chain planarity : 0.943	Side chain planarity : 1.513
Improper dihedral distribution : 0.572	Improper dihedral distribution : 1.156
Inside/Outside distribution : 1.237 (unusual)	Inside/Outside distribution : 1.276 (unusual)

We compared the 3D structure of SMS1 built in this work with our previous work [1]. The new model is a much more accurate three-dimensional structure

Sequence alignment

Table S2. Multiple Sequence alignment for the whole sequences of hSMS1 and hSMS2

CLUSTAL O(Version 1.2.4) multiple sequence alignment

```
SMS1      MKEVVYWSPKKVADWLLENAMPEYCEPLEHFTGQDLINLTQEDFKKPPLCRVSSDNGQRL      60

SMS2      ------------------------------------------------------------      0

SMS1      LDMIETLKMEHHLEAHKNGHANGHLNIGVDIPTPDGSFSIKIKPNGMPNGYR------KE      114

SMS2      MDIIETAKLEEHLENQPSDPTNTYARPAE--PVEEENKNGNGKPKSLSSGLRKGTKKYPD      58

         :*:*** *:*.*** : .. :* : . .   *. : . . : **:.: .* *      :

SMS1      MIKIPMPELERSQYPMEWGKTFLAFLYALSCFVLTTVMISVVHERVPPKEVQPPLPDTFF      174

SMS2      YIQIAMPTESRNKFPLEWWKTGIAFIYAVFNLVLTTVMITVVHERVPPKELSPPLPDKFF      118

           *:* **  .*.::*:** ** :**:**:  :*******:**********:.*****.**

SMS1      DHFNRVQWAFSICEINGMILVGLWLIQWLLLKYKSIISRRFFCIVGTLYLYRCITMYVTT      234

SMS2      DYIDRVKWAFSVSEINGIILVGLWITQWLFLRYKSIVGRRFCFIIGTLYLYRCITMYVTT      178

         *:::**:****:.****:******: ***:*:****:.***  *:***************

SMS1      LPVPGMHFNCSPKLFGDWEAQLRRIMKLIAGGGLSITGSHNMCGDYLYSGHTVMLTLTYL      294

SMS2      LPVPGMHFQCAPKLNGDSQAKVQRILRLISGGGLSITGSHILCGDFLFSGHTVTLTLTYL      238

         ********:*:*** ** :*:::**::**:********** :***:*:***** ******

SMS1      FIKEYSPRRLWWYHWICWLLSVVGIFCILLAHDHYTVDVVVAYYITTRLFWWYHTMANQQ      354

SMS2      FIKEYSPRHFWWYHLICWLLSAAGIICILVAHEHYTIDVIIAYYITTRLFWWYHSMANEK      298

         ********::**** ******..**:***:**:***:**::*************:***::

SMS1      VLKEASQMNLLARVWWYRPFQYFEKNVQGIVPRSYHWPFPWPVVHLSRQV-KYSRLVNDT      413

SMS2      NLKVSSQTNFLSRAWWFPIFYFFEKNVQGSIPCCFSWPLSWPPGCFKSSCKKYSRVQKIG      358
```

```
        ** :** *:*:*.**:  * :******* :* .: **: **   :. .  ****: :

SMS1    -------          413

SMS2    EDNEKST          365
```

* asterisk positions that have a single and fully conserved residue

: colon conservation between groups of strongly similar properties with a score greater than .5 on the PAM 250 matrix

. period conservation between groups of weakly similar properties with a score less than or equal to .5 on the PAM 250 matrix

Table S3: Prediction of sp|Q86VZ5|SMS1_HUMAN

ID	sp|Q86VZ5|SMS1_HUMAN			
FT	TOPO_DOM	1	134	CYTOPLASMIC.
FT	TRANSMEM	135	156	
FT	TOPO_DOM	157	181	NON CYTOPLASMIC.
FT	TRANSMEM	182	203	
FT	TOPO_DOM	204	214	CYTOPLASMIC.
FT	TRANSMEM	215	233	
FT	TOPO_DOM	234	279	NON CYTOPLASMIC.
FT	TRANSMEM	280	296	
FT	TOPO_DOM	297	304	CYTOPLASMIC.
FT	TRANSMEM	305	325	
FT	TOPO_DOM	326	330	NON CYTOPLASMIC.
FT	TRANSMEM	331	347	
FT	TOPO_DOM	348	413	CYTOPLASMIC.

Table S4: Prediction of sp|Q8NHU3|SMS2_HUMAN

ID	sp|Q8NHU3|SMS2_HUMAN			
FT	TOPO_DOM	1	78	CYTOPLASMIC.
FT	TRANSMEM	79	100	
FT	TOPO_DOM	101	125	NON CYTOPLASMIC.
FT	TRANSMEM	126	147	
FT	TOPO_DOM	148	158	CYTOPLASMIC.
FT	TRANSMEM	159	177	
FT	TOPO_DOM	178	216	NON CYTOPLASMIC.
FT	TRANSMEM	217	240	
FT	TOPO_DOM	241	248	CYTOPLASMIC.
FT	TRANSMEM	249	269	
FT	TOPO_DOM	270	274	NON CYTOPLASMIC.
FT	TRANSMEM	275	291	
FT	TOPO_DOM	292	365	CYTOPLASMIC.

Table S5. Multiple Sequence alignment for the transmembrane portion sequences of hSMS1 (residues from 131 to 353) and hSMS2 (residues from 75 to 297)

CLUSTAL O(1.2.4) multiple sequence alignment

```
SMS1_TM     ---EWGKTFLAFLYALSCFVLTTVMISVVHERVPPKEVQPPLPDTFFDHFNRVQWAFSIC      57

SMS2_TM     SMSEWWKTGIAFIYAVFNLVLTTVMITVVHERVPPKELSPPLPDKFFDYIDRVKWAFSVS      60

               ** ** :**:**:  :*******:**********:.*****.***:::**:****:.

SMS1_TM     EINGMILVGLWLIQWLLLKYKSIISRRFFCIVGTLYLYRCITMYVTTLPVPGMHFNCSPK      117

SMS2_TM     EINGIILVGLWITQWLFLRYKSIVGRRFCFIIGTLYLYRCITMYVTTLPVPGMHFQCAPK      120

            ****:******: ***:*:****:.***  *:***********************:*:**

SMS1_TM     LFGDWEAQLRRIMKLIAGGGLSITGSHNMCGDYLYSGHTVMLTLTYLFIKEYSPRRLWWY      177

SMS2_TM     LNGDSQAKVQRILRLISGGGLSITGSHILCGDFLFSGHTVTLTLTYLFIKEYSPRHFWWY      180

            * ** :*:::**::**:********** :***:*:***** **************::***

SMS1_TM     HWICWLLSVVGIFCILLAHDHYTVDVVVAYYITTRLFWWYHTMANQ      223

SMS2_TM     HLICWLLSAAGIICILVAHEHYTIDVIIAYYITTRLFWWYHSMANE      226

            * ******..**:***:**:***:**::*************:***:
```

* asterisk positions that have a single and fully conserved residue
: colon conservation between groups of strongly similar properties with a score greater than .5 on the PAM 250 matrix

. period conservation between groups of weakly similar properties with a score less than or equal to .5 on the PAM 250 matrix

Metadynamics Simulations

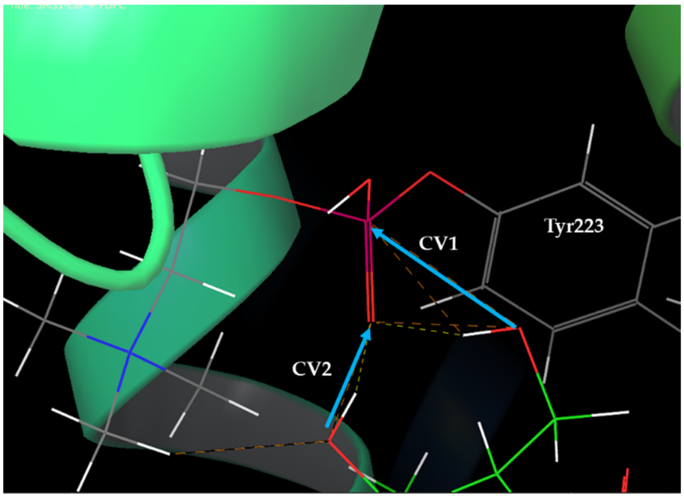

Figure S4. Atoms chosen as CV for metadynamics simulations for the SMS1 system.

The CV1 is the distance between the atom O5 of the Ceramide and the P1 of the modified tyrosine residue. The CV2 is the distance between the oxygen atom of the phosphate group and the hydroxyl group of the sphingosine chain.

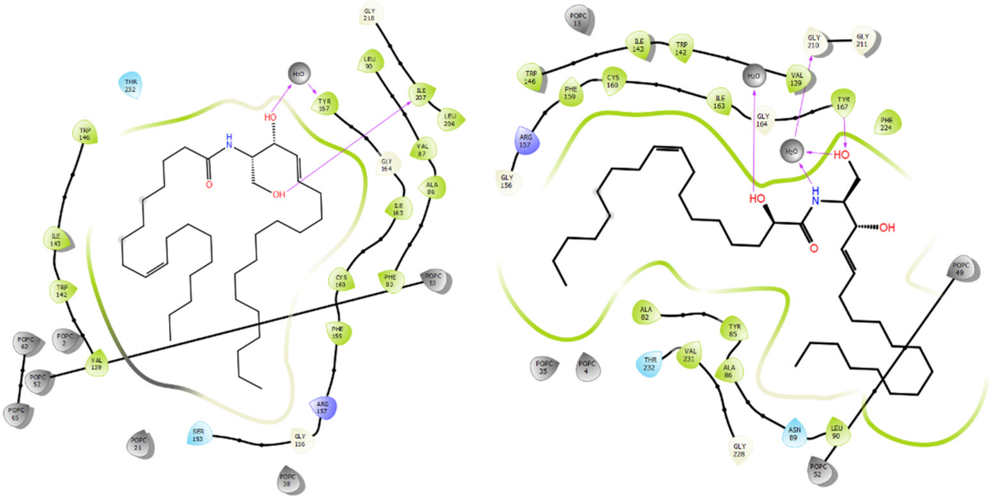

Figure S5. Ligand interaction diagram of Ceramide (on the left) and 2ROHCer (on the right) in complex with SMS2P. The pink arrows indicate the hydrogen bond that is established between the oxygen atoms of the ligand and the residues of the SMS2 protein.

Reference

1. Piotto, S.; Sessa, L.; Iannelli, P.; Concilio, S. Computational study on human sphingomyelin synthase 1 (hSMS1). *Biochimica et biophysica acta. Biomembranes* **2017**, *1859*, 1517-1525, doi:10.1016/j.bbamem.2017.04.004.
